# Chorea in Neuro-Behçet’s Disease

**DOI:** 10.7759/cureus.19039

**Published:** 2021-10-25

**Authors:** Mohamed Hamid, Kadira Adan, Amal Satte, Ahmed Bourazza

**Affiliations:** 1 Department of Neurology, Mohamed V Military Teaching Hospital, Mohamed V University, Rabat, MAR; 2 Department of Neurophysiology, Mohamed V Military Teaching Hospital, Mohamed V University, Rabat, MAR

**Keywords:** chorea, neuro-behcet’s disease, movement disorders, basal ganglia, cyclophosphamide

## Abstract

Behçet’s disease (BD) was described as a multisystemic recurrent inflammatory disorder of unknown cause comprising uveitis, skin lesions, recurrent genital, and oral ulcers. Involvement of the central nervous system in BD is about 10-25%. Chorea is defined as a hyperkinetic movement disorder, which can be caused by different etiologies. It was rarely mentioned in the literature as a manifestation of neuro-Behçet’s disease (NB). Radiological lesions are predominant in basal ganglia and periventricular white matter. The evolution of chorea in NB is variable in literature studies. We present a rare case of parenchymal NB with chorea.

## Introduction

Behçet’s disease (BD) is defined as a systemic inflammatory disorder with unknown etiology, characterized by uveitis, skin lesions, recurrent genital, and oral ulcers. It may be associated to vascular, intestinal, or neurological manifestations [1]. Implication of parenchymal or non-parenchymal central nervous system in BD is about 10-25% [2].

Movement disorders are extremely rare in neuro-Behçet’s disease (NB), with only a few cases mentioned in the literature. We report a patient presenting with chorea as an unusual manifestation of parenchymal NB.

## Case presentation

A 34-year-old right-handed male with a history of anterior uveitis and recurrent oral ulcers was diagnosed with BD four years ago. He was treated by cyclosporine and azathioprine, which were stopped 18 months ago. He had an attack with left hemiparesis nine months ago. Six days before his admission to the hospital, he reported progressive abnormal involuntary contractions in the right limbs, extended three days after to the left side.

Neurological examination revealed a conscious patient with apathy. He exhibited brief, irregular, and arrhythmic movements with large amplitude affecting the right limbs. Involuntary movements were predominant in the distal upper limb with flexion-extension movements of the fingers, forearm pronation-supination, and shoulder elevation-lowering. The lower limb movements were less intense causing disturbance of gait.

Examination of sensitivity and muscle strength was normal. There was a right muscular hypotonia. Deep tendon reflexes were present with the right Babinski sign. Cranial nerves were intact. Examination of other extra neurological systems was unremarkable.

The patient had no family history of abnormal movements disorder or Huntington’s disease. He had not received any dopaminergic antagonists and had no vascular risk factor.

Large paraclinical tests were performed and went all negative (hemogram, serum electrolytes, thyroid hormone, B12 and B9 seric levels, renal and hepatic tests). Anticardiolipin antibodies, antineutrophil cytoplasmic antibodies, and lupus anticoagulant were negative. HIV test was negative. Repeated peripheral blood smear revealed no acanthocytes. Urinary copper levels and serum ceruloplasmin were normal. Cerebrospinal fluid (CSF) was acellular with normal glucose and protein levels. Human leukocyte antigen (HLA) type was B 5101.

**Figure 1:**
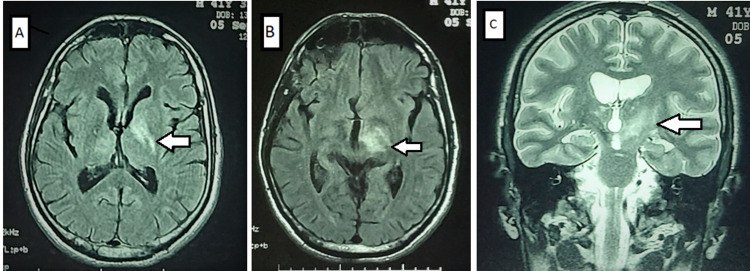
Brain MRI. Axial fluid-attenuated inversion recovery (FLAIR) sequences demonstrated the increased signal intensity of the left internal capsule (A) with the lesion of the left anteromedial thalamus (B). Coronal T2-weighted sequence (C) demonstrated the high-signal intensity of the left meso-diencephalic region. Location of cerebral lesions (white arrow).

Cerebral MRI showed high-signal intensity involving the left internal capsule, the left thalamus, and midbrain (Figure 1).

Our patient was treated by methylprednisolone bolus (1 g/day for five days) followed by administration of 1 g/day of intravenous (IV) methylprednisolone once a week for four weeks with 1 g IV cyclophosphamide administrated monthly. He received haloperidol for chorea. An oral tapering dose of steroid was performed with 40 mg/day and progressive degression up to a minimum dose of 5 mg/day. After 12 perfusions of cyclophosphamide, the patient was treated by azathioprine.

Evolution was favorable, with gradual improvement of chorea, and no complication or systemic relapse within 15 months follow-up.

## Discussion

Chorea is known as a hyperkinetic movement disorder characterized by irregular and brief contractions of limbs, trunk, or face. It represents irregular and unmodified spontaneous activity of the motor cerebral cortex [3]. Choreic movements can be generated by a large panel of etiologies, including vascular, genetic, metabolic, and pharmacological.

The movement disorder entity was rarely cited in the literature as a manifestation of NB [4,5]. In a study of Akman-Demir et al. [6], 200 patients with NB were analyzed, and only 6% of them had movement disorders.

The study of Benamour et al. [7] showed that 16.6% of 925 patients with BD had neurological manifestations, with only one case of chorea (0.006%).

The time between the onset of chorea in BD patients varied according to the literature. Chorea may appear at the onset of the disease or after 31 years of evolution of BD [4]. Our patient developed chorea four years after the onset of BD.

The few cases of chorea in NB reported in the literature revealed predominant radiological lesions in basal ganglia and periventricular white matter. Lesions were appearing in hyper signal on FLAIR sequence and T2-weighted images. FLAIR sequence showed more and clearer lesions than T2-weighted images [4,8]. Involvement of basal ganglia in NB is frequent, but chorea is still rare.

The radiological findings of some cases of chorea showed the existence of inflammatory lesions localized in the striatum, the thalamus, and exceptionally in the globus pallidus [3].

Thalamic lesions have been associated with various forms of movement disorders including athetosis, tremor, ballismus, hemi-dystonia, hemi-chorea, and chorea. The typical thalamic syndrome is characterized by choreiform movements, ataxic hemiparesis, superficial hemianesthesia with deep sensory disturbance, and severe spontaneous thalamic pain [9].

However, our patient had no sensory defects or ataxic hemiparesis, and he developed right hemi-chorea as the only initial manifestation of NB with left thalamic and midbrain lesion. The mechanism responsible for the interesting neurological features observed in our case is very difficult to speculate on in terms of its anatomical basis.

DeLong [10] proposed that hyperkinetic disorders such as chorea or ballismus highlights the loss of subthalamic nucleus control on the internal segment of the globus pallidus, followed by the disinhibition of thalamic neurons. In cases of NB, clinical characteristics, pathology, and cerebral MRI may not be concordant [6].

Histopathological features in NB disease corresponded to low-grade chronic lymphocytic meningo-encephalitis with widespread perivenular neutrophilic or lymphocytic and plasmocytic cuffing with multifocal necrotic foci. These abnormalities are found in the basal ganglia and brainstem, more than in the spinal cord [11,12].

In a study of Arber et al., three groups of patients with BD were analyzed based on the family history, HLA typing and clinical epidemiological data. Sixty three percentage of patients had HLA-B51 and 21% of them had HLA-B52 [13]. HLA-B51 was positive in our case.

In the case of Kuriwaka et al. [4], chorea was resolved by prednisolone, suggesting that autoimmune mechanism caused chorea. Our patient had mild progressive improvement in chorea after treatment by immunosuppressants and haloperidol, and it is hard to pronounce which one was effective.

## Conclusions

In conclusion, chorea is a movement disorder marked by involuntary spasmodic movements especially of the limbs and facial muscles. It can be generated by a vast list of causes, which is a real challenge for the clinician. Based on the present case, we suggest that NB should be included as one of the differential diagnosis of chorea, leading to prevent the disease progression by early treatment with immunosuppressants.
